# C-reactive protein influences the doctor’s degree of suspicion of pneumonia in primary care: a prospective observational study

**DOI:** 10.1080/13814788.2020.1852547

**Published:** 2021-01-05

**Authors:** Anna B. Moberg, Anna Ravell Jensen, Jakob Paues, Falk Magnus

**Affiliations:** a Kärna Primary Healthcare Centre, Linköping, Sweden; b Department of Health, Medicine and Caring Sciences, Linköping University, Linköping, Sweden; c Kungsgatan Primary Healthcare Centre, Linköping, Sweden; d Department of Biomedical and Clinical Sciences, Linköping University, Linköping, Sweden

**Keywords:** Community-acquired pneumonia, C-reactive protein, general practice, point of care tests, primary health care

## Abstract

**Background:**

In primary care, the diagnosis of pneumonia is often based on history and clinical examination alone. However, a previous study showed that the general practitioner’s degree of suspicion correlates well with findings on chest X-ray, when the C-reactive protein (CRP) value is known.

**Objectives:**

The present study aimed to investigate to what extent the physician’s degree of suspicion is affected by the CRP level when community-acquired pneumonia is suspected in primary care.

**Methods:**

A prospective observational study was conducted at five primary health care centres in Sweden between October 2015 and December 2017. Adult patients (*n* = 266) consulting their health care centre with symptoms of lower respiratory tract infection, where the physician suspected pneumonia, were included consecutively. Anamnestic information and findings from clinical examination were documented in a case report form. All patients were tested for CRP. The physicians rated their degree of suspicion as ‘unsure,’ ‘quite sure,’ and ‘sure’ before and after the CRP result.

**Results:**

The degree of suspicion of pneumonia changed in 69% of the cases; most often to a lower degree (40%). In 28% of the cases, there was no longer any suspicion of pneumonia after CRP.

**Conclusion:**

Our results indicate that CRP testing highly influences the physician’s degree of suspicion of pneumonia in primary care and that it seems to be of most value when not sure of the diagnosis.

KEY MESSAGES

- Testing for CRP in the assessment of pneumonia has high impact on the physician’s degree of suspicion of pneumonia.
- CRP testing seems to be most valuable when the GP is not sure of the diagnosis and hence could contribute excluding the diagnosis of pneumonia.

## Introduction

It is a challenge in primary care to correctly identify patients in need of antibiotics but also to avoid prescribing for self-limiting infections. Most patients consulting primary care for lower respiratory tract infections (LRTIs) have acute bronchitis, which is considered a self-limiting disease that should not be treated with antibiotics. However, some patients will have pneumonia, which is often of bacterial origin and may be lethal if not treated. In countries with a high antibiotic prescription rate, there are higher rates of antimicrobial resistance. Therefore, antibiotics should be used only for conditions where expected benefits exceed the risks, including the increase of antibiotic resistance on a society level [1,2].

Many studies have investigated different clinical findings and decision algorithms for the diagnosis of pneumonia, with varying results [3–6]. It has also been shown that clinical assessment differs between countries [7]. Guidelines regarding how to assess pneumonia in primary care vary. For example, C-reactive protein (CRP) testing is recommended in the initial judgement in some guidelines but not in others. Likewise, chest-X-ray (CXR) is recommended in the initial evaluation in some guidelines, whereas a more restrictive approach is used in others [8–12]. During bacterial infection, CRP level rapidly increases in serum due to inflammation and can be analysed in primary care as a point-of-care test. In Sweden, CRP is recommended when the diagnosis of pneumonia is unclear [12]. When interpreting the results, symptom duration should be considered as a factor, i.e. patients with symptoms of pneumonia should be treated if CRP is >100 mg/L at the visit or if CRP is <20 mg/L, and symptoms for more than one week. If CRP is <20 after 24 h of symptom duration, antibiotics should be refrained from [12]. Several studies have shown that CRP testing decreases antibiotic prescription rates [13–16], and improves the diagnostic accuracy and ability to assess infection risk level [17]. CXR is often considered ‘gold standard’ for the diagnosis of pneumonia, even though it is an imperfect gold standard and is not always available in primary care [18,19].

In a previous study, we showed that there was a strong correlation between the physician’s degree of suspicion of pneumonia and CXR results when the CRP value was known. For example, when physicians were sure of the diagnosis of pneumonia after examination, CXR was positive in 88% of the cases compared to only 28% when unsure [20]. However, which factors contribute to the degree of suspicion remains unclear. Until today little is known to what extent, and in what direction, CRP testing contributes in this respect.

The present study aimed to evaluate the impact of CRP-result on the physician’s degree of suspicion of pneumonia in primary care.

## Methods

### Study design

Four primary health care centres in the south of Sweden participated in this prospective observational study. All PHCCs were located less than 10 km from a hospital. Patients were included consecutively from 1 September 2015 to 31 December 2017. The chosen time interval of inclusion was adapted for another parallel stud to achieve the intended number of patients. Because of a slower inclusion rate than expected, one more health care centre was invited to participate from 1 December 2016.

### Selection of study participants

The physicians who included patients were either general practitioners (GPs), residents or interns. Consecutive patients ≥18 years, with lower respiratory tract symptoms for at least 24 h, who consulted one of the primary health care centres (PHCCs), and where the physician, after clinical examination, had some degree of suspicion of pneumonia, were included. The judgement of possible pneumonia was not specified any further, but was left to the physician. Exclusion criteria were living at a nursing home, self-reported pregnancy or diagnosis of chronic obstructive pulmonary disease (COPD) registered in the patient record.

### Measurements

Based on anamnesis and physical examination of the patient, the physicians rated their degree of suspicion of pneumonia into one of the same three categories used in our previous study: ‘unsure,’ ‘quite sure’ or ‘sure’ [20]. The suspicion degree was registered in a case report form (CRF). Patients then underwent CRP testing. After obtaining the CRP result the physicians rated their suspicion degree once again, this time with the added option ‘no longer suspicion of pneumonia.’ CRP was analysed using either Quickread go (Orion Diagnostica, Sweden) or Alere Afinion™ As100 Analyser. Information on age, sex, duration of symptoms, body temperature (measured by a digital ear thermometer), intake of antipyretics, abnormal chest sounds, and position of the physician was documented in the CRF. All patients included gave written informed consent. The study was approved by the Regional Ethical Review Board in Linköping(no. 2015/223-31).

### Statistics

The demographic characteristics and clinical data are presented as proportions and mean values or median values in skewed data. Pearson Chi-square test was used for crude group comparisons. To compare non-parametric data between more than two groups, we used Kruskal–Wallis test. Mann–Whitney *U*-test was used to compare medians. Logistic regression was performed to explore the association of different variables with the doctor’s degree of suspicion, dichotomised. Odds ratios were calculated with 95% confidence intervals. When missing data, the case was left out of the analyses. CRP results were categorised into subgroups (<20, 20–49, 50–99, and ≥100 mg/L) to analyse the influence of CRP on the suspicion degree. As different devices for CRP analyses were used, the devices had different upper limits, ranging from 160 to 200 mg/L, therefore all values above 160 and below 5 mg/L were set to 160 and 5 mg/L, respectively.

The sample size was calculated for the above parallel study and was based on the results from the previous study, described in the introduction, using similar outcome measures [20]. Statistical analyses were performed using IBM SPSS Statistics version 25 (IBM Corp., New York, USA). *P* values <0.05 were considered significant.

## Results

A total of 266 patients were recruited. One patient was excluded due to missing information on suspicion degree; thus, 265 patients were eligible for analyses. A temperature of 38 °C or more was measured in 52% of the patients. Somewhat less than half of the participants had used an antipyretic before the consultation. Symptom duration ranged from 1-64 days. The characteristics of the study population are presented in Table 1.

**Table 1.** Characteristics of the study population.

	Number of patients (*n* = 265) *n* (%)	Median (Interquartile range)	Data missing *n* (%)
Male	131 (49.4)		0
Female	134 (50.6)		
Age		53 (38;68)	
CRP		37 (11;84)	
Symptom duration		8 (6;14)	2
Smoking habits			6 (2.3)
Current smoker	23 (8.9)		
Ex-smoker	44 (17.0)		
Non-smoker	192 (74.1)		
Antipyretics	105 (42.5)		18 (6.8)
Abnormal chest sounds	133 (50.8)		3 (1.1)
Examined by			
General practitioner	164 (61.9)		1 (0.4)
Resident physician	53 (20.0)		
Interns	47 (17.8)		

### Association between CRP level and suspicion degree

**Figure 1.**
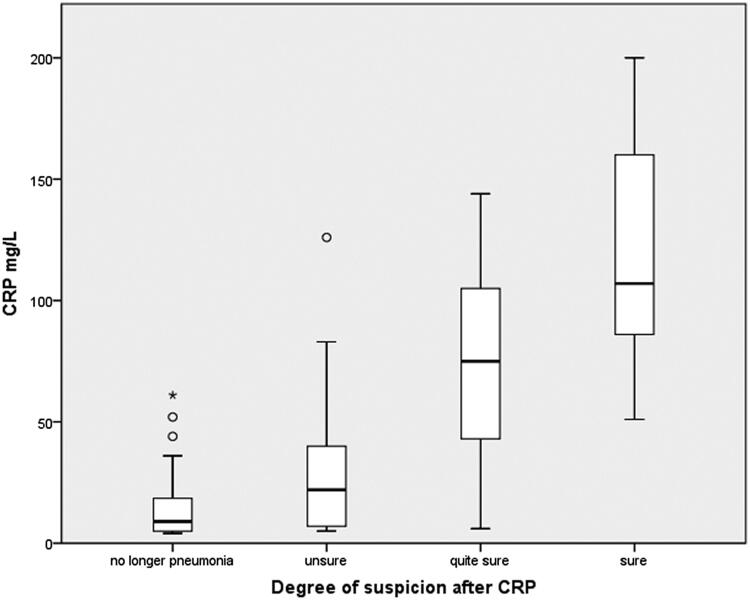
Median CRP levels in the different degrees of suspicion after CRP testing (*p <* 0.001*)*, Kruskal–Wallis test. Circles and asterisk represent outliers and extreme outliers, respectively (box and whisker plot).

The distribution of the degree of suspicion of pneumonia to CRP level is presented in Figure 1. There was a positive association between the degree of suspicion of pneumonia and CRP levels (*p* < 0.001). After CRP result, the physician was ‘sure’ of the diagnosis of pneumonia in 55 patients (median CRP 112 mg/L), ‘quite sure’ in 68 cases (median CRP, 63 mg/L) and unsure in 64 cases (median CRP, 23 mg/L). In 72 patients, there was no longer any suspicion of pneumonia according to the physician’s judgement after CRP testing (median CRP, 11 mg/L).

### Change in suspicion degree after CRP result

**Figure 2.**
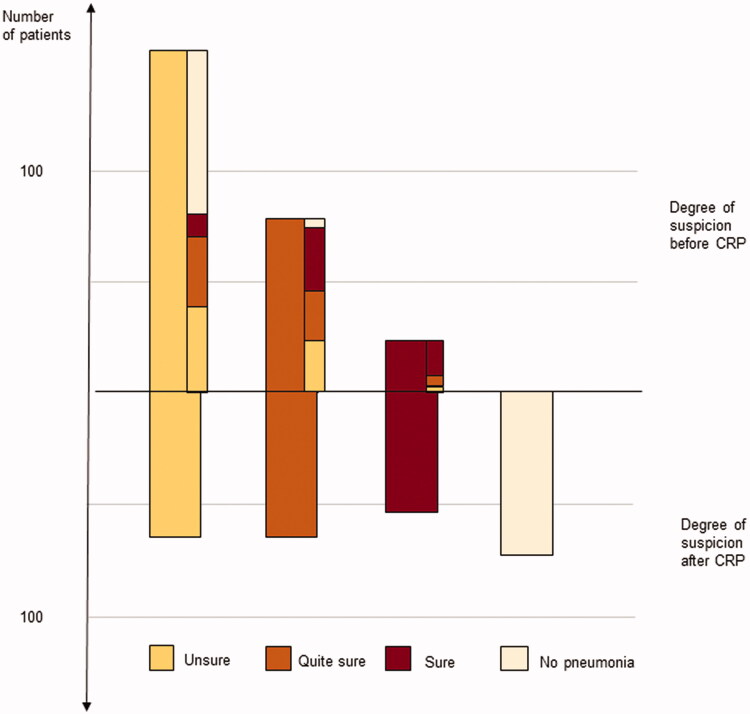
Distribution and shift in degree of suspicion before and after the CRP results. The narrow bars, in the upper part, represent the shift in degree of suspicion.

The physicians changed their degree of suspicion in 69% of the cases after CRP result. Of those, 40% shifted to a lower degree of suspicion, whereas 29% received a more substantial suspicion degree after CRP result. Overall, 28% of the patients were considered not having pneumonia after CRP analysis. The change in degree of suspicion before and after CRP is presented in Figure 2. The most-reported degree of suspicion before CRP was ‘unsure’ (*n* = 159). Of these, as many as 45% were regarded as ‘no pneumonia’ after CRP-testing, whereas the physician remained ‘unsure’ of the diagnosis in 26%. When the physician was ‘quite sure’ before CRP-testing, there was equal distribution of degree of suspicion remaining as ‘quite sure’ and changing to ‘unsure’ and a tendency to shift to a higher suspicion degree ( 38%) after CRP testing. The higher degree of suspicion before CRP, the less likely the suspicion degree shifted to ‘no longer suspicion of pneumonia’ (5% in the ‘quite sure’ group and 0% in the ‘sure’ group). There was no difference regarding the change in degree of suspicion between residents and specialists.

Median CRP for changing to a lower degree of suspicion was 11 mg/L (interquartile 5 and 25 mg/L) compared to 97 mg/L (interquartile 64 and 133 mg/L) when shifting to a higher degree of suspicion.

### Logistic regression

When a multiple logistic regression was performed on weak suspicion (including ‘unsure’) or strong suspicion (including ‘quite sure’ and ‘sure’), before CRP testing, as dependent and other findings as covariates, body temperature and abnormal chest sounds emerged as predictors of strong suspicion (OR 2.3 [95% CI 1.0–5.1] and OR 13 [95% CI 6.4–24], respectively. In a corresponding analysis with ‘any suspicion of pneumonia’ or ‘no longer pneumonia’ after CRP testing, as dependent variables, CRP emerged as a predictor of any suspicion of pneumonia and abnormal chest sounds remained associated with any suspicion of pneumonia (Table 2).

**Table 2.** Propensity of any degree of suspicion of pneumonia after CRP testing.

	Adjusted logistic regression *n* = 231 (missing 34)	
	*p* value	Odds ratio (95%CI)
Female	0.15	1.8 (0.81–4.2)
Ag*e* > 65 years	0.057	0.38 (0.14–1.07)
Symptom duration	0.39	1.0 (0.98–1.1)
Current smoker	0.73	1.3 (0.33–4.9)
Body temperatur*e* ≥ 38˚C	0.99	0.29 (0.29–3.4)
Abnormal chest sounds	<0.001	16 (6.4–38)
Intake of antipyretics	0.89	0.93 (0.39–2.2)
CR*p* ≥ 50 mg/L	<0.001	79 (19–335)

Nagelkerke *R*^2^ 0.571. Area under curve (AUC) 0.904.

## Discussion

### Main findings

The main finding in this study is that the physician’s degree of suspicion appears to be highly influenced by CRP result when pneumonia is suspected in patients consulting primary care for LRTI symptoms. To our knowledge, this has not been shown before and hence CRP testing is likely to be of relevance in the clinical judgement of suspected pneumonia in primary care especially when unsure of the diagnosis. When chest sounds are abnormal at auscultation, the physician seems to tend to suspect pneumonia both before and after CRP testing. From our study results it appears that when the clinical suspicion is strong, CRP does not contribute to the diagnosis as much as when in doubt. If the physician is unsure in the primary judgement, the outcome is more likely to remain unsure or lower to exclude pneumonia after CRP. The opposite tendency applies for higher degree of suspicion before CRP. Thirty-eight percent of the ‘quite sure’ group changed to ‘sure,’ and pneumonia was no longer suspected in only 5% from that group. None of the physicians in our study shifted from ‘sure’ to ‘no pneumonia’ after CRP testing. The median and interquartile range of CRP when shifting to a stronger or weaker degree of suspicion indicates adherence to guidelines.

This study has a pragmatic approach, notably not to evaluate the actual accuracy of the diagnostics of pneumonia but to illuminate the diagnostic considerations regarding CRP outcome, and we have no data on antibiotics prescribed or CXR result as gold standard.

### Strengths and limitations

The present study was performed in ordinary daily work at the PHCCs, and only patients for whom the physicians suspected pneumonia in the initial judgement were included, which we believe is a major strength of the study. Since the assessment of pneumonia was not specified any further, but was the physician’s individual and subjective judgement, different physicians probably would have made other decisions on the same patient. This is the real situation, since a clinical decision rule for pneumonia does not exist, which makes this study pragmatic. Because the study was executed in ordinary everyday settings and did not interfere with normal daily activities at the participating units, it truthfully reflects daily practice at PHCCs. It also highlights the daily challenges within primary care regarding how to arrive at a correct diagnosis, thereby counteracting potential over-prescription of antibiotics.

However, some limitations need to be highlighted. First, we cannot be sure that the participants in our study are representative of the Swedish population in general, regarding socioeconomic conditions and availability of health care. For example, in our cohort, the proportion of smokers was slightly lower (9%) than in the overall population in Sweden (10%) at the time of inclusion [21], possibly reflecting a sociodemographic difference. One explanation for the low percentage of smokers could be that patients with (known) COPD were excluded. Thus, the participants in our study might constitute a somewhat healthier group than the general population and there is a possibility that we believe more in CRP in this group. Furthermore, most patients were examined by a GP, suggesting a higher access level compared with the average Swedish PHCC. Another limitation is that there was no upper limit of symptom duration in the inclusion criteria, and there was a wide range of symptom duration, which could indicate that not all patients suffered from acute illness, or that they might have a chronic disease such as asthma as well. However, only nine out of 263 patients reported a symptom duration >30 days and when re-analysing data with these patients excluded, the overall shift in degree of suspicion did not change. Further, we do not know how many patients were not included and why. Inclusion was made consecutively by the primary care physicians as potential patients showed up at the participating primary health care centres. Nevertheless, it is likely that, due to working stress and shortness of time, several patients with some degree of suspicion of pneumonia were not included. Nor yet do we know if the patients diagnosed with pneumonia actually had pneumonia, as they were not all referred for CXR. However, in our previous study, 88% of the patients, where the physician’s degree of suspicion was ‘sure’ of the diagnosis of pneumonia, proved to have an infiltrate on CXR indicating pneumonia [20].

### Findings in relation to existing literature

It has been shown that GPs who use CRP testing in the diagnosis of pneumonia often overestimate the probability of radiographic pneumonia [22]. However, in our previous study, we found that the physician’s degree of suspicion correlates well to findings on CXR when CRP result is known. Among patients, for whom the degree of suspicion was rated ‘unsure’ in that study, 54% were prescribed antibiotics [20]. If we assume that 54% of the patients in the present study, where the physician was unsure initially, would have been treated this way and that those where the physician excluded the diagnosis of pneumonia were left without an antibiotic prescription, 86 instead of 35 patients would have been prescribed antibiotics. This is in line with earlier studies where it has been shown that CRP testing in respiratory tract infections (RTIs) reduces the antibiotic prescription rate [13,15,16,23,24] and that it plays a vital role in the decision on whether to prescribe antibiotics or not [25]. Moreover, meta-analyses showed that when CRP is added to the assessment of pneumonia in primary care, the diagnostic discrimination improved but in patients with intermediate risk at the decision making the challenge remains [26]. However, most previous studies were not based on patients with suspected pneumonia, but either on all RTIs or all LRTIs. In an earlier study of patients with acute cough, it was shown that CRP influenced a GP’s decision on whether to prescribe antibiotics or not, but physicians with an already restrictive antibiotic prescription rate were not affected. Whether the GPs in the present study already had a restrictive approach, with or without CRP testing, is unknown. Since CRP was introduced in Swedish primary care in the 1990s, the GPs are comfortable with the test, although this may not be the same in other countries and might influence the generalizability of the results.

A Dutch study suggested that patients with milder LRTI symptoms and CRP <20 mg/L are at low risk for pneumonia [3]. This is in concordance with the Swedish guidelines mentioned above, where pneumonia is suggested to be excluded when CRP is less than 20 mg/L. However, in another study CRP level did not prove to be a reliable measure to ban pneumonia in primary care [27].

Other factors, for example progression of symptoms over time, have also been shown to predict community-acquired pneumonia in primary care [28].

## Conclusion

In conclusion, although it is likely that other factors may affect the decision making, e.g. anamnestic details, clinical examination findings, or the impression of the patient’s general health condition, this study suggests that CRP result highly influences the degree of suspicion of pneumonia. Further CRP testing seems to be most valuable when the GP is not sure of the diagnosis and hence could contribute to exclude the diagnosis of pneumonia and thereby might keep the prescriptions of antibiotics restrictive and purposeful. It would be of further interest to investigate how, and to what extent, other clinical information, as mentioned above, affects the physician’s degree of suspicion to understand the essence of clinical decision making better. Further, it would be valuable with a study investigating the degree of suspicion to CRP and antibiotic prescription.
